# Medical resource utilization and the associated costs of asthma in China: a 1-year retrospective study

**DOI:** 10.1186/s12890-023-02685-0

**Published:** 2023-11-22

**Authors:** Xueer Yang, Tiantian Zhang, Xuanyi Yang, Jie Jiang, Yuwen He, Pei Wang

**Affiliations:** 1 College of Pharmacy, Jinan University, Guangzhou, 510632 China; 2 International Cooperative Laboratory of Traditional Chinese Medicine Modernization and Innovative Drug Development of Ministry of Education (MOE) of China, Jinan University, Guangzhou, 510632 China; 3 Guangzhou Huabo Biopharmaceutical Research Institute, Guangzhou, 510010 China; 4 Department of Pharmacy, The First Affiliated Hospital of Guangzhou Medical University, 151 Yanjiang Road, Guangzhou, 510120 China; 5 School of Public Health, Fudan University, 130 Dong An Road, Shanghai, 200032 China; 6 Key Lab of Health Technology Assessment, National Health Commission of the People’s Republic of China (Fudan University), Shanghai, 200032 China

**Keywords:** Asthma, Costs and cost analysis, Economic burden

## Abstract

**Background:**

Despite evidence that severe and poorly controlled asthma are associated with more clinical unmet needs and intensive utilization of healthcare resources, limited data is available on severe asthma expenditure in China. The study aimed to assess Medical Resource Utilization (MRU) costs of asthma and explore the cost drivers in order to better understand the economic burden of the Chinese population suffered from asthma.

**Methods:**

A retrospective analysis was conducted using Chinese sampled national claim database. Patients aged 6 years and above with primary diagnosis of asthma and asthma-related medical visit/hospitalization during 2015 were included. Medication was used as a proxy per the GINA and China guideline to identify asthma severity (i.e. mild, moderate, and severe). multiple linear regression model was conducted to identify MRU costs drivers.

**Results:**

7,254 patients diagnosed with asthma were included: 4,529 (62.4%), 2,200 (30.3%), and 525 (7.2%) had mild, moderate, and severe asthma, respectively. On average, each severe patient spent 6,782 Chinese Yuan (CNY) on asthma treatment and had 57.0% hospitalization rate during the year, 3.9- and 4.4-fold of the average of overall population (P < 0.001 for both). The proportion of patients experiencing exacerbation significantly higher in the severe asthma population (66.5%; P < 0.001) compared to mild (30.0%) and moderate (16.8%) groups. In subgroup with 1,660 samples had annual consecutive data, severe patients had annual cost of CNY 8,314 and 52.2% hospitalization rate. 13% of severe patients who had frequent severe exacerbation (≥ 2 events) experienced the highest annual average cost CNY 23,037, P < 0.001) whereas children aged from 6 to 14 with a lower annual cost of CNY 1,094.2, 1,660.2 and 3,020.2 for mild, moderate, and severe patients respectively. The multiple model identified degree of severity, control status, complications, age, and live region as independent drivers of MRU costs.

**Conclusions:**

Chinese asthma patients bear heavy economic burden. Severe asthma associated with higher MRU (mainly from hospitalization) and costs compared with mild to moderate asthma in China. More efforts should be devoted to the control of the disease severity and complication as the main drivers of asthma cost.

**Supplementary Information:**

The online version contains supplementary material available at 10.1186/s12890-023-02685-0.

## Introduction

Asthma is a common chronic airway disease worldwide. In spite of its lower prevalence in China (adults older than 20 of 4.2% and children under 14 of 2.6%) compared to some other countries, it has been increasing significantly during the last decades [1–3]. For instance, according to the China Asthma and Risk Factor Epidemiologic (CARE) survey, the prevalence among individuals aged > 14 years dramatically increased by one to two folds in Beijing (91.23%) and Shanghai (178.0%) in the past 10 years [4]. In 2019, the age standardized DALYs rate of asthma ranks 8th among 369 diseases in China [5]. Hence, asthma is increasingly becoming a serious public health concern in China.

The economic burden of asthma often associates with high consumption of medical resources. Medical resources utilization (MRU) involves emergency room (ER) and outpatient visits, hospital admission, as well as medications incurred during the visits and admission. A number of studies with different designs (e.g. retrospective and prospective, cross-sectional) and perspectives (e.g. societal perspective and health care perspective) have been performed in different countries to quantify the economic impact of asthma and cost structure [6–12]. Although the asthma costs showed high variability which is not surprising, hospitalization and medications costs were found to be the important contributors of asthma costs. Many studies have also consistently identified asthma severity is the most important drivers of the costs [13, 14]. A number of factors including control and exacerbation status, comorbidity conditions, age, and gender were also detected.

Despite the studies contributed to the understanding of economic impact of asthma, the issue in China still needs further clarification. Several hospital-based, cross-sectional studies have assessed general asthma costs and economic burden of specific population such as occupational asthma patients and asthma inpatients. Some studies also explored the cost drivers at different regions in China [15–18]. However, limited data is available on asthma, especially severe asthma costs including outpatient and inpatient expenses and cost drivers at national level. Hence, the aims of study were to assess medical resource utilization (MRU) systematically and comprehensively, associated costs and the relationships between MRU costs and a set of potential costs drivers using Chinese sampled national claim database. Particular emphases were given to the association of asthma severity with the MRU and costs as a minority of severe asthma patients consumed a majority of medical resources according to international findings.

## Method

### Study design and data source

It was a retrospective study using data from China Health Insurance Research Association (CHIRA), which is the only available database at national level representing overall MRU and related costs of Chinese patients covered by the mainstream health insurance. The study was conducted and has been granted an exemption from requiring ethics approval by the Institutional Review Board of School of Public Health, Fudan University. The data were collected from the local insurance offices/centers of selected areas including 4 Municipalities (i.e. Beijing, Shanghai, Tianjin, Chongqing) under the central government, all the capital cities, and 1 ~ 2 prefecture-level cities within each province. All the beneficiaries listed via descending ages are subject to random equidistant sampling for recording medicine use and treatment over a full year; and sample ratio for those patients with medical records was 2% and 5% for municipalities and capital cities, and prefecture-level cities, respectively.

The database included patients’ consecutive inpatient and outpatient visit claims including resource usage and medical resource utilization (MRU) costs as well as their sociodemographic, and treatment expenditure information.

### Study population

Patients with consecutive medical records, primary diagnosis of asthma [ICD 10 code: J45 (asthma)/J46 (persistent asthma)] from 01 January to 31 December 2015 were used as the sampling frame. As in most cased, both prescribing and dispensing occur at the same hospital visit and patients cannot re-fill the prescription by their own, the asthma diagnosis, rather than prescription records, is used as inclusive criteria. The inclusion and exclusion criteria were with primary diagnosis of asthma and persistent asthma; and without diagnosis of cancer, active lung disease such as COPD, chronic bronchitis, emphysema, respectively.

As there is no longitudinal follow-up on patients beyond the one-year timeframe and it is impossible to distinguish between prevalent and incident patients, we proposed to use annual sub-sample (patients from 01 January to 28 February 2015) to estimate accurate annual HRU and medical cost.

As medication and clinical practice on children asthma patients are often distinct from adults and children are more vulnerable with less available therapies and more unmet clinical need, children aged from 6 to 14 in the whole sample and sub-sample were also part of the sampling frame.

### Assessment of clinical characteristics including asthma severity, exacerbation, control status, and existence of severe complication

Medication was used as a proxy per the Global Initiative for asthma (GINA) and China guideline [1, 19] to identify asthma severity (i.e. mild, moderate, and severe). Since dosage is not available from the database, as well as prescription practice in China reflected after data cleaning, clinical expert opinion was also considered. Mild asthma is the patients having one of the following treatments: only SABA, or only ICS, or controller/ controller combination except ICS (i.e. LABA, LAMA, Theophylline). Moderate asthma is the patients on double combination with ICS, such as ICS + LABA/LAMA, or ICS + LTRA, or ICS + theophylline. Severe asthma is the patients on triple combination with ICS.

Exacerbation was defined as: hospital admission or emergency room (ER) visit (severe exacerbation), or outpatient visit with an OCS prescription order within 7 days of outpatient visit (non-severe exacerbation). Uncontrolled asthma was defined as:≥1 any exacerbation and/or treatment step-up with treatment add-ons. Events occurring within 14 days will be considered the same exacerbation.

Existence of asthma complication is defined as having one of the following conditions related to asthma exacerbation which may lead to unfavorable prognosis: infection, shock, pneumothorax, disseminated intravascular coagulation, mechanical ventilation, respiratory failure, respiratory or sudden cardiac arrest, and sudden death [20, 21].

### Medical resource utilization and the associated costs

Medical resources utilization associated with asthma were retrieved from the dataset, comprising information of outpatient visits, ER visits, hospitalization, medication and tests. The associated costs such as costs per patient/visit/admission were also recorded in the dataset. Based on these, the asthma MRU and costs by disease severity, average costs for specific asthma events such as exacerbation, hospitalization and ER visit were calculated.

All costs in the study were expressed in Chinese Yuan (CNY); the US dollar equivalent can be calculated using the mean of 2015 exchange rates: US$1 = CNY 6.2284.

### Data analysis

The study variables were summarized using descriptive statistics: frequencies and percentage for categorical variables; means and standard deviations, for continuous variables. Their differences according to asthma severity were compared using appropriate methods. In detail, Chi-square tests/Fisher-exact tests were used for categorical variables. Shapiro-Wilk normality test was adopted to assess the normality of distribution of continuous variables. Student’s t tests/ANOVA tests were used for those achieving normal distribution; Mann-Whitney U /Kruskal-Wallis tests were for the others.

Total MRU and associated costs were present according to asthma severity. The costs according to demographic and disease variables including age, gender, region, insurance type, complication prevalence, control level, exacerbation status, and hospitalization were also provided. Mann-Whitney U /Kruskal-Wallis tests were used to compare the costs according to different levels within each variable in order to identify cost drivers.

The association of asthma severity with total costs was assessed using two multiple regression models. The first model adopted the logarithm of costs as dependent variable and adjusted for the factors mentioned above except for exacerbation status and hospitalization. They were excluded because they closely correlated with control level. As there may be a modifying effect of control level on severity. The second model included all the variables in the first model plus the interaction between control level and severity to test the modifying effect of control level on severity. A three-level ordinal variable representing by two dummy variables was therefore created to indicate different effects of poor control on moderate and severe severity. An F test of variance was used to compare the goodness of fit of two models in order to identify whether the use of interaction improved model performance.

All analyses were performed using STATA 14.0.

## Results

### Participants’ characteristics

A total of 7,254 patients diagnosed with asthma during 2015 met the inclusion criteria. The average patient age was 48.6 with adult covered the majority. 6.6% patients were children under 12 years. Female patients exceeded the half (53.7%), 85.4% participants were Urban Employee Basic Medical Insurance (UEBMI) insured and 86.1% lived in east China.

Among the patients, 4,529 (62.4%), 2,200 (30.3%), and 525 (7.2%) had mild, moderate, and severe asthma, respectively. Their characteristics according to severity are described in Table 1.

**Table 1:** Sociodemographic and clinical characteristics of study population according to asthma severity

	Mild (n = 4,529)	Moderate (n = 2,200)	Severe* (n = 525)	Total (n = 7,254)	p-Value
**Age, years (SD)**	**49.1 (19.5)**	**46.8 (17.9)**	51.7 (17.4)	48.6 (18.9)	0.0001
** 6–11**	327 (7.2%)	**129 (5.9%)**	20 (3.8%)	476 (6.6%)	
** 12–17**	58 (1.3%)	**39 (1.8%)**	3 (0.6%)	100 (1.4%)	
18–30	363 (8.0%)	197 (9.0%)	39 (7.4%)	599 (8.3%)	
31–45	1,028 (22.7%)	631 (28.7%)	116 (22.1%)	1,775 (24.5%)	
46–60	1,400 (30.9%)	694 (31.6%)	181 (34.5%)	2,275 (31.4%)	
>60	1,353 (29.9%)	510 (23.2%)	166 (31.6%)	2,029 (28.0%)	
**Gender (%, male)**	2,045 (45.2%)	**1,093 (49.7%)**	222 (42.3%)	3,360 (46.3%)	< 0.0001
**Region (%)**					
East	**3,884 (85.8%)**	**2,010 (91.4%)**	352 (67.1%)	6,246 (86.1%)	< 0.0001
Middle	**336 (7.4%)**	**95 (4.3%)**	103 (19.6%)	534 (7.4%)	
West	**309 (6.8))**	**95 (4.3%)**	70 (13.3%)	474 (6.5%)	
**Frequency of hospital visits** (%)**†**					< 0.0001
Tertiary	**3,610(32.1%)**	**3,988 (59.5%)**	671 (64.2%)	8,269 (43.5%)	
Secondary	**2,193(19.5%)**	**1,399 (20.9%)**	263 (25.2%)	3,855 (20.3%)	
Primary	**5,451(48.4%)**	**1,315 (19.6%)**	111 (10.6%)	6,877 (36.2%)	
Unspecified	**3(0%)**	**1 (0%)**	0 (0%)	4 (0%)	
**Insurance type** (%)					< 0.0001
Urban Employee Basic Medical Insurance (UEBMI)	**3,823 (84.4%)**	**1,957 (89.0%)**	415 (79.1%)	6,195 (85.4%)	
Urban Resident Basic Medical Insurance (URBMI)	**706 (15.6%)**	**243 (11.1%)**	110 (21.0%)	1,059 (14.6%)	
**Complications(%, yes)**	**155 (3.4%)**	**47 (2.1%)**	31 (5.9%)	233 (3.2%)	< 0.0001
**Exacerbation (%, yes)**	**1,360 (30.0%)**	**369 (16.8%)**	349 (66.5%)	2,078 (28.7%)	< 0.0001
Frequency (SD, all patients)	**0.5(1.6)**	**0.3(1.1)**	0.8(0.7)	0.5(1.5)	0.0001
Frequency (SD, patients who had the Exacerbation)	**1.7(2.6)**	**1.7(2.4)**	1.2(0.6)	1.6(2.4)	0.0001
**Severe exacerbation (%, yes)**	**1312(29.0%)**	**353(16.1%)**	349(66.5%)	2014(27.8%)	< 0.0001
Frequency (SD, all patients)	**0.5(1.6)**	**0.3(1.1)**	0.8(0.7)	0.4(1.4)	0.0001
Frequency (SD, patients who had the Severe exacerbation)	**1.7(2.6)**	**1.7(2.4)**	1.2(0.6)	1.6(2.4)	0.0002
**Control level (%, poor)**	**1,612 (35.6%)**	**483 (22.0%)**	384 (67.5%)	2449 (33.8%)	< 0.0001

*Reference group. †Each patient may go to hospital (either same or different levels of hospital) repeatedly, resulting in the frequency larger than the number of patients. SD: standard deviation. Age was compared in analysis of variance test; Chi-square tests/Fisher-exact tests were used for the comparison of other variables. *P* values indicated overall significance of each comparison. Two separate significance testes were also used to compare mild group vs. severe group, and moderate group vs. severe group, respectively. Bold indicates values that are significantly different from the reference group (i.e., severe group). Complications were defined as having one of the following conditions: infection, shock, pneumothorax, disseminated intravascular coagulation, mechanical ventilation, respiratory failure, respiratory or sudden cardiac arrest, and sudden death; Exacerbation was defined as: hospital admission or emergency room (ER) visit, or outpatient visit with an OCS prescription order within 7 days of outpatient visit; Poor control level was defined as: ≥1 any exacerbation or treatment step-up

Mild to moderate asthma patients were more likely to be younger, reside in eastern region, visit primary hospital, and shielded by UEBMI. The proportion of patients experiencing exacerbation during the year was significantly higher in the severe asthma population (66.5%; P < 0.001) compared to mild (30.0%) and moderate (16.8%) groups. 67.5% severe patients failed in disease control, compared with 22% in moderate and 35.6% in mild (P < 0.001). Severe patient more tends to encounter complications leading to unfavorable prognosis (P < 0.001).

### Medical resource utilization by asthma severity

Severe asthma was significantly related to higher hospitalization rate, more and longer hospitalization and ER days, as well as higher rates of medication use and medical test (Table 2).

**Table 2:** Medical resource utilization by asthma severity

	Mild	Moderate	Severe*	Total	p-Value
**Outpatient visits**					
Rate (%)	**3,798 (83.9%)**	**2,002(91.0%)**	234(44.6%)	6,034(83.2%)	0.000
Frequency (SD, all patients )	**2.2 (3.2)**	**2.8 (3.7)**	1.3 (2.4)	2.3 (3.3)	0.0001
Frequency (SD, patients who had the visits)	2.6(3.3)	3.1(3.8)	2.8(3.0)	2.8(3.5)	0.0001
Duration (day)	1.0(0.0)	1.0(0.0)	1.0(0.0)	1.0(0.0)	1.0000
**Emergency room visits**					
Rate (%)	**493 (10.9%)**	146 (6.6%)	39 (7.4%)	678 (9.4%)	0.000
Frequency (SD, all patients)	**0.2 (0.7)**	0.1(0.9)	0.1(0.3)	0.2 (0.7)	0.0123
Frequency (SD, patients who had the visits)	1.6 (1.3)	1.9 (3.1)	1.1 (0.3)	1.6 (1.8)	0.1626
Duration (day)	**1.1(0.8)**	**1.1 (1.1)**	2.9 (3.7)	1.2 (1.2)	0.0296
**Hospital admission**					
Rate (%)	**487 (10.8%)**	**172 (7.8%)**	299 (57.0%)	958 (13.2%)	0.000
Frequency (SD, all patients)	**0.1 (0.4)**	**0.1 (0.3)**	0.6 (0.7)	0.1 (0.4)	0.0001
Frequency (SD, patients who had the admission)	1.1 (0.4)	1.1 (0.4)	1.1 (0.6)	1.1 (0.5)	0.8623
Duration (day)	**9.9 (4.7)**	**10.1 (6.7)**	11.5 (5.9)	10.4 (5.5)	0.0001
**Medication**					
Utilization rate of OCS (%)	**117 (2.6%)**	**33 (1.5%)**	110 (21.0%)	260 (3.6%)	0.0000
Utilization rate of ICS + LABA (%)	**278 (6.1%)**	**1,902 (86.5%)**	436 (83.1%)	2,616 (36.1%)	0.0000
**Diagnosis**					
Detection rate of total IgE (%)	**49 (1.1%)**	**22 (1.0%)**	43 (8.2%)	114 (1.6%)	0.000

*Reference group. SD: standard deviation. OCS: oral corticosteroid use; ICS + LABA: combination inhaled corticosteroid/long-acting beta agonist; IgE: Immunoglobulin E. Frequency was compared in analysis of variance test; rate was compared in Chi-square tests/Fisher-exact tests. *P* values indicated overall significance of each comparison. Two separate significance testes were also used to compare mild group vs. severe group, and moderate group vs. severe group, respectively. Bold indicates values that are significantly different from the reference level

For example, 57.0% severe asthma patients experienced hospitalization for average 11.5 days, while only 10.8% and 7.8% admission rate in mild and moderate group respectively (P < 0.0001). ICS + LABA was used in more than 80% moderate to severe patients. 21% severe patients used OCS. On the other hand, mild and moderate asthma were associated with higher outpatient rate and frequency. Despite the fact that around 70% asthma is allergic as reported [22], very few (1.6%) patients had IgE test, with 8.2% in severe group as the highest (P = 0.000).

### Medical resource utilization costs by asthma severity

Total MRU costs ranged from CNY 0.1 to CNY 209,356, with the mean and median being CNY 1,734 and CNY 571, respectively. The mean and standard deviation of total and the three specific costs (i.e. outpatient visits, emergency room visits, and hospitalization) were present stratifying by asthma severity in Table 3.

**Table 3:** Costs of medical resource utilization by asthma severity

	Mild	Moderate	Severe*	Total	p-Value
**Total costs (SD)**	**1,136.0 (2,340.5)**	**1,760.4 (5,369.6)**	6,782.0 (10,324.4)	1,734.0 (4,683.0)	0.0001
Medication	**760.8 (1,327.9)**	**1,323.2 (2,987.4)**	3,896 (6,181.5)	1,158.2 (2,686.4)	0.0001
Lab test and medical imaging	**166.6 (620.8)**	**205.4 (1,604.5)**	1,308.1(1,810.8)	261.0 (1,159.2)	0.0001
Total IgE detection	**0.7(7.7)**	**0.8(14.3)**	5.2(23.2)	1.0(11.8)	0.0243
Mechanical ventilation	**2.4(102.9)**	**0.1(5.6)**	29.2(469.8)	3.6(150.4)	0.8881
**Outpatient visits costs (SD)**	532.4 (909.2)	**1,082.3 (1,745.4)**	611.4 (1,256.0)	704.9 (1,271.3)	0.0001
Medication	470.0 (846.6)	**973.2 (1,608.5)**	533.9 (1,158.1)	627.3 (1,175.2)	0.0001
Lab test and medical imaging	23.7 (112.5)	39.5 (187.7)	24.3 (96.6)	28.5 (139.0)	0.8460
Total IgE detection	0.1(2.2)	0.0(0.0)	0.0(0.0)	0.0(1.7)	0.9944
**Emergency room visits costs (SD)**	**67.5 (342.2)**	**55.6 (465.2)**	178.1 (1,118.5)	71.9 (479.6)	0.0154
Medication	**44.0 (221.0)**	**37.3 (286.2)**	87.6 (574.1)	45.1 (281.6)	0.0146
Lab test and medical imaging	**9.5 (93.9)**	**8.0 (130.7)**	49.2 (337.9)	11.9 (138.0)	0.3356
Total IgE detection	**0.0(1.0)**	**0.0(0.0)**	0.2(3.0)	0.0(1.1)	0.9907
Mechanical ventilation	**0.2(9.3)**	**0.0(0.0)**	0.0(0.0)	0.1(7.4)	0.9944
**Hospital admission costs (SD)**	**536.1 (2,213.4)**	**622.4 (5,149.7)**	5,992.6 (10,604.9)	957.2 (4,604.8)	0.0001
Medication	**246.7 (1,077.6)**	**312.6 (2,589.7)**	3,274.4 (6,340.5)	485.8 (2,504.1)	0.0001
Lab test and medical imaging	**133.4 (604.5)**	**157.9 (1,586.6)**	1,234.5 (1,821.8)	220.5 (1,145.2)	0.0001
Total IgE detection	**0.6(7.3)**	**0.8(14.3)**	5.0(23.1)	1.0(11.6)	0.0313
Mechanical ventilation	**2.2(102.5)**	**0.1(5.6)**	29.2(469.8)	3.5(150.2)	0.8733
Hospital bed fee	**21.9 (97.5)**	**21.1 (163.3)**	191.6 (272.4)	33.9 (146.00)	0.0001

Cost data are presented as mean in Chinese Yuan (CNY); the US dollar equivalent can be calculated using the mean of 2015 exchange rates: US$1 = CNY 6.2284. *Reference group. SD: standard deviation. Costs were compared in Kruskal-Wallis; *P* values indicated overall significance of each comparison. Two separate significance testes were also used to compare mild group vs. severe group, and moderate group vs. severe group, respectively

Total costs increased with degree of severity (CNY 1,130 for mild, CNY 1,760 for moderate, and CNY 6,782 for severe). Severe patients associated with significantly higher annual and event medical costs (P = 0.0001). The high annual cost was mainly attributed to hospitalization costs. Hospitalization covered 80% of severe asthma annual medical costs, with CNY 5,992.6 per patient per year, significantly higher than CNY 536.1 in mild and CNY 622.4 in moderate group (P = 0.0001). The average medical cost for each severe exacerbation was CNY 7,992 for severe patients, 3-fold of the overall patient population average. Aggregately, 7% patients consumed 28% of total asthma medical expenditure.

### Correlates of medical resource use costs

Total costs of asthma patients according to their demographic and disease characteristics are displayed in Table 4.

**Table 4:** Costs of medical resource utilization by sociodemographic and clinical characteristics

Variable	Mean Costs	SD		*P*
**Age group (years)**				0.0001
** 6–11**	1,122.2	1,618.7		
** 12–17**	1,348.1	1,702.5		
** 18–30**	1,032.0	1,837.0		
** 31–45**	1,363.7	5,505.3		
** 46–60**	1,720.6	3,385.3		
** >60**	2,442.8	6,037.9		
**Gender**				0.1675
** Male**	1,735.3	5,791.9		
** Female**	1,732.8	3,451.6		
**Region**				0.0001
** East**	1,401.5	3,788.1		
** Middle**	5,217.6	5,158.4		
** West**	2,190.4	10,073.1		
**Insurance type**				< 0.0001
Urban Employee Basic Medical Insurance	1,640.2	4,804.2		
Urban Resident Basic Medical Insurance	2,282.7	3,855.5		
**Complications (yes)**	5,902.3	14,139.4		< 0.0001
**Complications (no)**	1,595.7	3,931.3		
**Controlled**	717.0	1,163.5		< 0.0001
**Uncontrolled**	3,729.4	7,503.7		
**Exacerbation ≥ 2 (yes)**	4,264.4	8,275.0		< 0.0001
**Exacerbation ≥ 2 (no)**	1,559.9	4,273.9		
**Hospitalization (yes)**	7,373.3	10,796.6		< 0.0001
**Hospitalization (no)**	875.9	1,403.7		

Cost data are presented as in Chinese Yuan (CNY); the US dollar equivalent can be calculated using the mean of 2015 exchange rates: US$1 = CNY 6.2284. SD: standard deviation. *P* values were based on Mann-Whitney U /Kruskal-Wallis tests. Complications were defined as having one of the following conditions: infection, shock, pneumothorax, disseminated intravascular coagulation, mechanical ventilation, respiratory failure, respiratory or sudden cardiac arrest, and sudden death; Exacerbation was defined as: hospital admission or emergency room (ER) visit, or outpatient visit with an OCS prescription order within 7 days of outpatient visit; Poor control level was defined as: ≥1 any exacerbation or treatment step-up.

Significantly higher costs were observed for greater severity, uncontrolled asthma patients, frequent exacerbation (≥ 2 events), existence of complications and hospitalization. Age and region were also significantly associated with costs. There was no significant difference in total costs between female and male. Asthma patients with and without hospitalization had the highest and lowest total costs, respectively (i.e. CNY 7,373 vs. CNY 876).

### Multiple analysis of medical resource use costs

According to the multiple model without interaction terms, the coefficients for moderate and severe asthma were 0.85 (95% confidence interval [CI]: 0.80, 0.91) and 1.57 (95% CI: 1.47, 1.67), respectively. These indicated that they associated with a 135% and a 382% increase in MRU costs, assuming invariant other variables. The model also indicated that significantly higher MRU cost were related to poor control level, presence of complications, older age, and living in middle region (Table 5).

**Table 5:** multiple regression analysis on the logarithm of medical resource utilization costs

	Coefficients	p-Value	95% Confidence Interval
**Intercept**	4.663	< 0.0001	4.537 to 4.790
**Age**	0.006	< 0.0001	0.004 to 0.007
**Male(vs. female)**	-0.032	0.214	-0.082 to 0.018
**Region(vs. west)**			
East	0.710	< 0.0001	0.606 to 0.814
Middle	1.637	< 0.0001	0.467 to 0.752
**Insurance** (vs. Urban Resident Basic Medical Insurance)			
Urban Employee Basic Medical Insurance	-0.062	0.120	-0.141 to 0.016
**Asthma severity(vs. mild)**			
Moderate	0.854	< 0.0001	0.799 to 0.910
Severe	1.572	< 0.0001	1.473 to 1.672
**Control status(vs. well controlled)**	1.138	< 0.0001	1.078 to 1.197
**Complications(vs.no)**	0.610	< 0.0001	0.467 to 0.752
**Adjusted R** ^**2**^	0.400		

Complications were defined as having one of the following conditions: infection, shock, pneumothorax, disseminated intravascular coagulation, mechanical ventilation, respiratory failure, respiratory or sudden cardiac arrest, and sudden death; Exacerbation was defined as: hospital admission or emergency room (ER) visit, or outpatient visit with an OCS prescription order within 7 days of outpatient visit; Poor control level was defined as: ≥1 any exacerbation or treatment step-up.

Inclusion of interaction terms did not improve model performance according to the F test (p > 0.05); and none of them reached statistical significance.

### Subgroup analysis on annual medical resource utilization costs by asthma severity

During Jan 1 to Feb 28, 2015, 1,660 eligible patients were included: 996 (60%), 595 (35.8%), and 69 (4.2%) had mild, moderate, and severe asthma, respectively (Appendix 1). In the subgroup with complete yearly cost data, the mean MRU costs were CNY 2,015; severe patients had annual costs of CNY 8,313 and 52.2% hospitalization rate, which were 4.8- and 6.7-fold of mild to moderate (P < 0.001 for both) (Appendix 2&3).

Further exploration conducted to identify extreme MRU cost and its driver among severe asthma patients. 10% of severe patients had annual expenditure exceeding CNY 17,706 and 3% exceeding CNY 30,000. 13% severe patients who had frequent severe exacerbation (≥ 2 events) experienced the highest annual average cost of CNY 23,037.2 (P = 0.0002).

### Potential unmet needs among children aged from 6 to 14 years old

Among children aged from 6 to 14 years old, 4% were server with 31.8% experienced hospitalization, significantly higher than mild (P = 0.001) to moderate (P = 0.011). Despite the possible side effect, corticosteroids were widely used in children with severe asthma: 77.3% used ICS, 31.8% used OCS and 40.9% used systematic corticosteroids, which was significantly high (P = 0.001). The severe exacerbation rate was lower in children aged from 6 to 14 years old compared to those above 14, but still significant higher in severe than mild (P = 0.014) to moderate asthma (P = 0.03) (Appendix 4).

Using annual sub-sample to estimate accurate annual medical costs, the overall MRU costs were lower for children with CNY 1,094.2, 1,660.2 and 3,020.2 for mild, moderate, and severe patients respectively.

## Discussion

To the best of our knowledge, it is the first study with the largest sample size that comprehensively assessed MRU and related costs in varied populations, regions and clinical settings as well as identified the main drivers of the MRU costs in Chinese asthma patients [15–18]. The mean costs for full sample and subsample were $278 and $324, respectively. The costs in China are much lower compared to those in western countries and several Asian countries even considering inflation rates [10, 22–26]. For example, Eric et al. estimated that annual asthma per capita healthcare costs in Singapore were $930, and Kim et al. revealed that the annual direct costs of adult asthmatic patients in Korea were $1,324 [10, 26] It should be noted that various factors could affect the country difference in costs (e.g. healthcare and insurance systems; clinical and socio-demographic characteristics of asthma patients, and economic status).

Stratification by severity clearly showed that it positively associated with overall MRU cost, which was also confirmed by the multiple regression model. Moreover, the costs increased disproportionately for severe asthma: 3.9-fold of the average costs of overall sample ($1,089 vs. $278). As a result, a small proportion of severe asthma patients (i.e. 7.5%) accounted for approximately 28% of MRU costs in the study. These findings are generally in line with previous studies [10, 11, 27–29]. For instance, direct costs were substantially higher in severe asthma group in the above-mentioned study conducted in Korea ($2,214 vs. $978 for moderate and $871 for mild) [10]. Also, 7.8% of severe patients accounted for 20.6% of total costs according to findings from Antonicelli et al. [11].

Among the three kinds of MRU costs, hospitalization and ER visits costs constituted the largest and smallest component for the whole sample, respectively. Medication costs contribute a large proportion of MRU costs. These findings were similar with prior studies [13, 24, 30–32] Meanwhile, hospitalization costs for mild and moderate asthma were not higher or even lower than outpatient visits costs; while it was disproportionately high for severe asthma comprising about 90% of MRU costs in the group. It may not be surprising as the proportion of poor asthma control was also very high in the group (67.4%) and hospitalization normally occurs when the management of asthma has failed. Indeed, multiple regression model revealed that poor control status, independently of severity, was associated with statistically significantly higher MRU costs in the study. Moreover, it also showed that the effect of poor control on MRU costs in each severity category was similar as the interaction term was not statistically significant. Although this imply that better disease control would help to decrease the economic burden (particularly the burden of hospitalization) across all severities, considering disproportionate burden and unmet needs of severe asthma, related costs could be substantially reduced by improving management of severe asthma.

The model also indicated that age, region and complications were significant drivers of MRU costs. These factors, together with severity, explained a considerable proportion of the variance of MRU costs (R^2^ = 0.40). Age has long been recognized as a risk factor of asthma costs, though the direction of association conflicts [13]. We found MRU costs increased with age which may be due to older asthmatic patients are more likely to induce hospitalization and need more medications. The association between presence of complications and MRU costs was consistent with prior studies and expected as additional medical resources would be needed to treat the complications resulting in increased costs [13, 24]. An interesting finding is asthma patients living in middle region associated with higher MRU costs than their counterparts in eastern and western regions, while eastern region has the best economic development level in China. We may be unable to explain the finding as the variables included in the dataset were mainly related to the assessment of MRU and its costs, but not for the identification of costs drivers. Hence, we had no information regarding a number of potential drivers such as comorbidity status, patient compliance, income and education levels, and smoking status. Future studies systematically assessing the correlations between MRU costs and its factors are therefore warranted. Meanwhile, the relationship between gender and MRU costs was not supported according to the model, though several studies reported that female were found to have higher direct costs [33, 34]. The study in Korea did not detect the association yet [10].

In the study, we also investigated the MRU and costs for asthma patients aged from 6 to 14 years old in order to deepen the understanding. Similar to adults, severe patients were faced with high exacerbation rate, poor control and high costs. The mean costs of children for full sample and subsample were CNY 1,148 and CNY 1,339, which were much higher than that of CNY 525 in a previous similar research in China with a younger definition and inclusion criteria of children (0–14 years old) [16]. The reason behind the cost difference between two researches was speculated that patients at ages of 0–5 could only be treated with basic therapies, resulting in a less MRU costs of the latter research [16]. Although the annual MRU costs were lower for children probably due to limited pediatric medication choice, we observed high utilization of corticosteroids, especially OCS and systematic corticosteroids for severe patients, which may lead to great adverse effect to children development [35–38]. This reflected the unmet needs for severe pediatric asthma management despite the small patient group. However, the sample size for the two kinds of patients may not be big enough to provide an accurate estimation. Hence, future studies based on a larger sample of children or severe asthma patients are warranted.

### Limitations

The dataset and sampling strategy used in the study could have the ability to draw a representative sample of asthmatic patients in urban China. Some limitations of the study should also be noted. 1)Our data was retrieved from 2015 rather than recent data. First, as a national database, the process of data collection, cleaning, and analysis is very time-consuming. Second, the acquisition of such a large database requires authorization, and the sensitive respondent information in the data also requires time for data masking. Hence, there is a time lag between the availability of data and the current report; 2) Medication was used as proxy to define disease severity and control level, which could be different from the GINA step definition. Meanwhile, the data used is the medical insurance database without the required clinical data used in GINA. Thus, we cannot classify the patients in terms of the clinical parameters. In addition, this kind of definition of severity classification has also been widely used in previous studies [39, 40]; 3) Since only health resources used in the hospital was captured with ICD diagnose, no test or treatment results (incl. death) could be obtained to link with the therapy; 4) Sampling by CHIRA database is done on a yearly basis, but the sampled population can be different every year. Non-consecutive data were available currently, thus it is impossible to set up washout period for incident patients or observe data out of the nature year; 5) Data from retail pharmacy was not available, so self-treatment data may be missed. However, since most asthma medications need to be prescribed by doctors, no great impact is expected; 6) Due to data limitation, we were unable to assess a number of important factors described above. The findings cannot be generalized to asthmatic patients in rural areas; 7) Our research did not mention that the co-morbidities associated with asthma. As we stated above, the dataset did not include the clinical parameters such as co-morbidities. Thus, we cannot assess its impacts on MRU and costs.

In conclusion, the study showed that MRU and the associated costs of severe asthma patients were disproportionately higher than those with mild and moderate asthma in China, which was mainly due to hospitalization costs. Exacerbation and poor control of asthma were detected as important drivers of MRU costs, especially in severe asthma. Presence of complications, older age, and living in middle region were also significant drivers of the increased MRU costs in China. The findings are useful in identifying effective strategies and making sound health policies to relieve the economic burden of asthma.

### Electronic supplementary material

Below is the link to the electronic supplementary material.

**Additional file 1:** Supplementary Table 1. Sociodemographic and clinical characteristics of annual sub-sample according to asthma severity. Supplementary Table 2. Medical resource utilization of annual sub-sample by asthma severity. Supplementary Table 3. Costs of medical resource utilization of annual sub-sample by asthma severity. Supplementary Table 4. Medical resource utilization by children under 14 years.

## Data Availability

The data that support the findings of this study are available from China Health Insurance Research Association (CHIRA) but restrictions apply to the availability of these data, which were used under license for the current study, and so are not publicly available. Data are however available from the authors upon reasonable request and with permission of China Health Insurance Research Association (CHIRA).

## Abbreviations

CHIRA: China Health Insurance Research Association
MRU: Medical Resource Utilization
GINA: Global Initiative for Asthma
CNY: Chinese Yuan
CARE: China Asthma and Risk Factor Epidemiologic
DALYs: Disability Adjusted of Life Years
ER: Emergency Room
COPD: Chronic Obstructive Pulmonary Disease
SABA: Short-acting Beta2 agonist
ICS: Inhaled corticosteroid
LABA: Long-acting Beta2 agonist
LAMA: Long-acting muscarine anticholinergic
LTRA: Leukotriene receptor antagonist
OCS: Oral corticosteroid
UEBMI: Urban Employee Basic Medical Insurance
ICD: International Classification of Diseases

## References

[1] [Guidelines for bronchial asthma prevent and management(2020 edition) Asthma group of Chinese Throacic Society]. Zhonghua Jie He He Hu Xi Za Zhi. 2020;43(12):1023-4810.3760/cma.j.cn112147-20200618-00721. 10.3760/cma.j.cn112147-20200618-00721 33333637

[2] Huang K, Yang T, Xu J, Yang L, Zhao J, Zhang X (2019). Prevalence, risk factors, and management of asthma in China: a national cross-sectional study. Lancet.

[3] Shu W, Li ML, Li ZA, Hu Y-f (2020). [Meta-analysis of asthma prevalence of children aged 0–14 in surveillance cities of China]. Zhonghua Yu Fang Yi Xue Za Zhi.

[4] Lin J, Wang W, Chen P, Zhou X, Wan H, Yin K (2018). Prevalence and risk factors of asthma in mainland China: the CARE study. Respir Med.

[5] Liu M, Gan H, Lin Y, Lin R, Xue M, Zhang T, et al. Prevalence and disability-adjusted Life Year Rates of Asthma in China: findings from the GBD Study 2019 of the G20. Int J Environ Res Public Health. 2022;19(22). 10.3390/ijerph192214663. 10.3390/ijerph192214663 PMC9690014 36429381

[6] Alefan Q, Nawasrah A, Almomani B, Al-Issa ET (2022). Direct Medical cost of Pediatric Asthma in Jordan: a cost-of-illness Retrospective Cohort Study. Value Health Reg Issues.

[7] Lopez-Tiro J, Contreras-Contreras A, Rodriguez-Arellano ME, Costa-Urrutia P (2022). Economic burden of severe asthma treatment: a real-life study. World Allergy Organ J.

[8] Pugliese FR, Guglielmelli E, Angelini D, Cicchini C, Castaldo E, Di Girolamo F (2020). Pharmacoeconomic management of patient with severe asthma in the Emergency Department: retrospective multicentric and cost of illness study. Eur Rev Med Pharmacol Sci.

[9] Roche N, Nadif R, Fabry-Vendrand C, Pillot L, Thabut G, Teissier C (2022). Asthma burden according to treatment steps in the french population-based cohort CONSTANCES. Respir Med.

[10] Kim SH, Kim TW, Kwon JW, Kang HR, Lee YW, Kim TB (2012). Economic costs for adult asthmatics according to severity and control status in korean tertiary hospitals. J Asthma.

[11] Antonicelli L, Bucca C, Neri M, De Benedetto F, Sabbatani P, Bonifazi F (2004). Asthma severity and medical resource utilisation. Eur Respir J.

[12] Schwenkglenks M, Lowy A, Anderhub H, Szucs TD (2003). Costs of asthma in a cohort of swiss adults: associations with exacerbation status and severity. Value Health.

[13] Bahadori K, Doyle-Waters MM, Marra C, Lynd L, Alasaly K, Swiston J (2009). Economic burden of asthma: a systematic review. BMC Pulm Med.

[14] Nunes C, Pereira AM, Morais-Almeida M (2017). Asthma costs and social impact. Asthma Res Pract.

[15] Huang MJ, Zhang JB, Liu J, Sun DY, Chen H (2020). [Analysis of direct economic burden of occupational asthma]. Zhonghua Lao Dong Wei Sheng Zhi Ye Bing Za Zhi.

[16] Wu P, Xu B, Shen A, He Z, Zhang CJP, Ming WK (2020). The economic burden of medical treatment of children with asthma in China. BMC Pediatr.

[17] Ding B, DiBonaventura M, Karlsson N, Ling X (2017). A cross-sectional assessment of the prevalence and burden of mild asthma in urban China using the 2010, 2012, and 2013 China National Health and Wellness surveys. J Asthma.

[18] Lin JT, Xing B, Tang HP, Yang L, Yuan YD, Gu YH (2017). [A multi-center retrospective study of clinical characteristics and hospitalization costs of patients hospitalized for asthma exacerbation in China during 2013–2014]. Zhonghua Jie He He Hu Xi Za Zhi.

[19] Reddel HK, Bacharier LB, Bateman ED, Brightling CE, Brusselle GG, Buhl R (2022). Global Initiative for Asthma Strategy 2021: executive Summary and Rationale for Key Changes. Respirology.

[20] Oe K, Araki T, Ogawa H, Nakashima A, Sato K (2010). Fatal cytomegalovirus infection with CD4 + T-lymphocytopenia during corticosteroid therapy for bronchial asthma. J Infect Chemother.

[21] D’Amato G, Vitale C, Lanza M, Sanduzzi A, Molino A, Mormile M (2016). Near fatal asthma: treatment and prevention. Eur Ann Allergy Clin Immunol.

[22] Chan PW, Hussain S, Ghani NH, Debruyne JA, Liam CK (2002). The direct cost of treating bronchial asthma in a teaching hospital in Malaysia. Southeast Asian J Trop Med Public Health.

[23] Chuesakoolvanich K (2007). Cost of hospitalizing asthma patients in a regional hospital in Thailand. Respirology.

[24] Bavbek S, Malhan S, Mungan D, Misirligil Z, Erdinc M, Gemicioglu B (2021). Economic burden of severe asthma in Turkey: a cost of illness study from payer perspective. Eur Ann Allergy Clin Immunol.

[25] Barbosa JP, Ferreira-Magalhaes M, Sa-Sousa A, Azevedo LF, Fonseca JA (2017). Cost of asthma in portuguese adults: a population-based, cost-of-illness study. Rev Port Pneumol (2006).

[26] Finkelstein EA, Lau E, Doble B, Ong B, Koh MS. Economic burden of asthma in Singapore. BMJ Open Respir Res. 2021;8(1). 10.1136/bmjresp-2020-000654. 10.1136/bmjresp-2020-000654 PMC7978329 33737309

[27] Nagase H, Adachi M, Matsunaga K, Yoshida A, Okoba T, Hayashi N (2020). Prevalence, disease burden, and treatment reality of patients with severe, uncontrolled asthma in Japan. Allergol Int.

[28] Cisternas MG, Blanc PD, Yen IH, Katz PP, Earnest G, Eisner MD (2003). A comprehensive study of the direct and indirect costs of adult asthma. J Allergy Clin Immunol.

[29] Lin HC, Kao S, Wen HC, Wu CS, Chung CL (2005). Length of stay and costs for asthma patients by hospital characteristics–a five-year population-based analysis. J Asthma.

[30] Zannetos S, Zachariadou T, Zachariades A, Georgiou A, Talias MA (2017). The economic burden of adult asthma in Cyprus; a prevalence-based cost of illness study. BMC Public Health.

[31] Yuan J, Lu ZK, Xiong X, Jiang B. Lowering drug prices and enhancing pharmaceutical affordability: an analysis of the national volume-based procurement (NVBP) effect in China. BMJ Glob Health. 2021;6(9). 10.1136/bmjgh-2021-005519. 10.1136/bmjgh-2021-005519 PMC8438819 34518200

[32] Mao W, Jiang H, Mossialos E, Chen W. Improving access to medicines: lessons from 10 years of drug reforms in China, 2009–2020. BMJ Glob Health. 2022;7(11). 10.1136/bmjgh-2022-009916. 10.1136/bmjgh-2022-009916 PMC9639057 36332928

[33] Serra-Batlles J, Plaza V, Morejon E, Comella A, Brugues J (1998). Costs of asthma according to the degree of severity. Eur Respir J.

[34] Accordini S, Bugiani M, Arossa W, Gerzeli S, Marinoni A, Olivieri M (2006). Poor control increases the economic cost of asthma. A multicentre population-based study. Int Arch Allergy Immunol.

[35] Raissy HH, Kelly HW (2017). Benefits and risks of long-term Asthma Management in Children: where are we heading?. Drug Saf.

[36] Loke YK, Blanco P, Thavarajah M, Wilson AM (2015). Impact of inhaled corticosteroids on growth in children with asthma: systematic review and Meta-analysis. PLoS ONE.

[37] Nuyen B, Weinreb RN, Robbins SL (2017). Steroid-induced glaucoma in the pediatric population. J AAPOS.

[38] de Queiroz Mendonca C, de Souza CP, Martins-Filho PR, Viana SS, Leal BC, Cipolotti R (2014). Steroid-induced ocular hypertensive response in children and adolescents with acute lymphoblastic leukemia and non-hodgkin lymphoma. Pediatr Blood Cancer.

[39] Pollack M, Gandhi H, Tkacz J, Lanz M, Lugogo N, Gilbert I (2022). The use of short-acting bronchodilators and cost burden of asthma across Global Initiative for Asthma-based severity levels: insights from a large US commercial and managed Medicaid population. J Manag Care Spec Pharm.

[40] Jacob C, Bechtel B, Engel S, Kardos P, Linder R, Braun S (2016). Healthcare costs and resource utilization of asthma in Germany: a claims data analysis. Eur J Health Econ.

