# Supplementary material for: Medical resource utilization and the associated costs of asthma in China: a 1-year retrospective study
Source: BMC Pulm Med. 2023 Nov 22;23:463. doi: 10.1186/s12890-023-02685-0 (PMC10666355; doi:10.1186/s12890-023-02685-0)
Supplement: Supplementary file 1 — Additional file 1: Supplementary Table 1. Sociodemographic and clinical characteristics of annual sub-sample according to asthma severity. Supplementary Table 2. Medical resource utilization of annual sub-sample by asthma severity. Supplementary Table 3. Costs of medical resource utilization of annual sub-sample by asthma severity. Supplementary Table 4. Medical resource utilization by children under 14 years. [file 12890_2023_2685_MOESM1_ESM.doc]

**Supplementary Table 1. Sociodemographic and clinical characteristics of annual sub-sample** **according to asthma severity**

|  | **Mild**  **(n=996)** | **Moderate**  **(n=595)** | **Severe***  **(n=69)** | **Total**  **(n=1,660)** | **p-Value** |
| --- | --- | --- | --- | --- | --- |
| **Age, years (SD)** | 50.8 (20.3) | **46.9 (19.2)** | 51.7 (17.4) | 49.5 (19.8) | 0.0001 |
| **6-11** | 84 (8.4%) | 45 (7.6%) | 2 (2.9%) | 131 (7.9%) | 0.002 |
| **12-17** | 9 (0.9%) | 20 (3.4%) | 0 (0%) | 29 (1.8%) |  |
| **≥18** | 903 (90.7%) | 530 (89.1%) | 67 (97.1%) | 1,500 (90.4%) |  |
| **Gender (%, male)** | 488 (49.0%) | 309 (51.9%) | 34 (49.3%) | 831 (50.1%) | 0.521 |
| **Region (%)** |  |  |  |  |  |
| East | 876 (88.0%) | **561 (94.3%)** | 59 (67.1%) | 1,496 (90.1%) | 0.0001 |
| Middle | 52 (5.2%) | **14 (2.4%)** | 5 (19.6%) | 71 (4.3%) |  |
| West | 68 (6.8%） | **20 (3.4%)** | 5(13.3%) | 93 (5.6%) |  |
| **Frequency of hospital visits** (%)**†** |  |  |  |  | <0.0001 |
| Tertiary | **1,349(33.0%)** | 1,879 (65.2%) | 163 (65.7%) | 3,391 (43.5%) |  |
| Secondary | **662 (16.2%)** | 501 (17.4%) | 36 (14.5%) | 1,199 (20.3%) |  |
| Primary | **2,082(50.9%)** | 500 (17.4%) | 49 (19.8%) | 2,631 (36.2%) |  |
| **Insurance type** (%) |  |  |  |  | 0.911 |
| Urban Employee Basic Medical Insurance (UEBMI) | 848 (85.1%) | 510 (85.7%) | 58 (84.1%) | 1,416 (85.3%) |  |
| Urban Resident Basic Medical Insurance (URBMI) | 148 (14.9%) | 243 (14.3%) | 11 (15.9%) | 244 (14.7%) |  |
| **Complications(%, yes)** | 47 (4.7%) | 16 (2.7%) | 3 (4.4%) | 66 (4.0%) | 0.132 |
| **Exacerbation (%, yes)** | **308 (30.9%)** | **97 (16.3%)** | 42 (60.9%) | 447 (26.9%) | <0.0001 |
| **Control level (%, poor)** | **407 (40.9%)** | **154 (25.9%)** | 44 (63.8%) | 605 (36.5%) | <0.0001 |

*****Reference group. †Each patient may go to hospital (either same or different levels of hospital) repeatedly, resulting in the frequency larger than the number of patients. SD: standard deviation. Age was compared in analysis of variance test; Chi-square tests/Fisher-exact tests were used for the comparison of other variables. *P* values indicated overall significance of each comparison. Two separate significance testes were also used to compare mild group vs. severe group, and moderate group vs. severe group, respectively. Bold indicates values that are significantly different from the reference group (i.e., severe group). Complications were defined as having one of the following conditions: infection, shock, pneumothorax, disseminated intravascular coagulation, mechanical ventilation, respiratory failure, respiratory or sudden cardiac arrest, and sudden death; Exacerbation was defined as: hospital admission or emergency room (ER) visit, or outpatient visit with an OCS prescription order within 7 days of outpatient visit; Poor control level was defined as: ≥1 any exacerbation or treatment step-up.

**Supplementary Table 2. Medical resource utilization of annual sub-sample by asthma severity**

|  | **Mild** | **Moderate** | **Severe*** | **Total** | **p-Value** |
| --- | --- | --- | --- | --- | --- |
| **Outpatient visits** |  |  |  |  |  |
| Rate (%) | **929 (93.3%)** | **580 (97.5%)** | 57 (82.6%) | 1,566(84.3%) | 0.000 |
| Frequency (SD, all patients ) | **35.2 (38.9)** | **39.1 (46.7)** | 22.0 (26.9) | 36.0 (41.6) | 0.0001 |
| Frequency (SD, patients who had the visits) | 37.7(39.1) | 40.1(46.8) | 26.6 (27.4) | 38.2(41.8) | 0.0349 |
| **Emergency room visits** |  |  |  |  |  |
| Rate (%) | **404 (40.6%)** | 222 (37.3%) | 19 (27.5%) | 645 (38.9%) | 0.0630 |
| Frequency (SD, all patients) | 1.7 (4.1) | 1.5 (3.6) | 1.6(4.8) | 1.6 (3.9) | 0.1155 |
| Frequency (SD, patients who had the visits) | 4.3 (5.5) | 3.9 (4.9) | 5.8 (7.9) | 4.2 (5.4) | 0.4399 |
| Duration (day) | 1.1(0.9) | **1.0 (0.2)** | 1.2 (1.9) | 1.1 (0.8) | 0.9101 |
| **Hospital admission** |  |  |  |  |  |
| Rate (%) | **190 (19.1%)** | **73 (12.3%)** | 36 (52.2%) | 299 (18.0%) | 0.000 |
| Frequency (SD, all patients) | **0.5 (2.1)** | **0.3 (1.9)** | 0.9 (1.2) | 0.4 (2.0) | 0.0001 |
| Frequency (SD, patients who had the admission) | 2.5 (4.2) | 2.6 (4.7) | 1.7 (1.2) | 2.5 (4.1) | 0.7588 |
| Duration (day) | **6.9 (6.5)** | **5.8 (7.3)** | 11.0 (4.7) | 7.0 (6.7) | 0.0001 |
| **Medication** |  |  |  |  |  |
| Utilization rate of OCS (%) | **68 (6.8%)** | **30 (5.0%)** | 19 (27.5%) | 117 (7.1%) | 0.0000 |
| Utilization rate of ICS+LABA (%) | **145 (14.6%)** | 549 (92.3%) | 65 (94.2%) | 759 (45.7%) | 0.0000 |
| **Diagnosis** |  |  |  |  |  |
| Detection rate of total IgE (%) | **45 (4.5%)** | **26 (4.4%)** | 11 (15.9%) | 82 (4.9%) | 0.000 |

*****Reference group. SD: standard deviation. OCS: oral corticosteroid use; ICS+LABA: combination inhaled corticosteroid/long-acting beta agonist; IgE: Immunoglobulin E. Frequency was compared in analysis of variance test; rate was compared in Chi-square tests/Fisher-exact tests. *P* values indicated overall significance of each comparison. Two separate significance testes were also used to compare mild group vs. severe group, and moderate group vs. severe group, respectively. Bold indicates values that are significantly different from the reference level.

**Supplementary Table 3. Costs of medical resource utilization of annual sub-sample by asthma severity**

|  | **Mild** | **Moderate** | **Severe*** | **Total** | **p-Value** |
| --- | --- | --- | --- | --- | --- |
| **Total costs (SD)** | **1,506.9 (2889.2)** | **2,136.7 (3175.5)** | 8,313.8 (9,559.5) | 2015.6 (3,764.7) | 0.0001 |
| Medication | **1,115.0 (1,777.0)** | **1,784.4 (2,544.1)** | 5,210.4 (6,779.0) | 1,525.1.2 (2,604.8) | 0.0001 |
| Lab test and medical imaging | **182.0 (907.9)** | **151.0 (616.8)** | 1,563.0 (2,013.6) | 228.3 (935.0) | 0.0001 |
| **Outpatient visits costs (SD)** | 896.7 (1,429.6) | 1,663.6 (2,489.4) | 1,237.4 (2,108.3) | 1,185.7 (1,938.8) | 0.0001 |
| Medication | 806.6 (1,336.5) | 1,516.3 (2,323.4) | 1,147.9 (2,057.8) | 1075.2 (1,175.2) | 0.0001 |
| Lab test and medical imaging | 35.0 (158.2) | 44.6 (191.7) | 31.7 (120.8) | 38.3 (169.7) | 0.8996 |
| **Emergency room visits costs (SD)** | 96.7 (447.3) | 55.4 (324.2) | 127.7 (842.9) | 83.2 (432.7) | 0.2867 |
| Medication | 63.5 (296.7) | 45.6 (255.7) | 47.5 (244.7) | 56.4 (280.6) | 0.2902 |
| Lab test and medical imaging | **11.8 (103.0)** | **4.0 (46.6)** | 60.6 (448.9) | 11.1 (124.6) | 0.6044 |
| **Hospital admission costs (SD)** | **513.5 (2,470.3)** | **417.7 (2,137.8)** | 6,948.7 (10,269.3) | 746.7 (3,360.1) | 0.0001 |
| Medication | **244.9 (1,173.3)** | **222.5 (1,206.7)** | 4,015.1 (7,158.7) | 393.6 (2,004.2) | 0.0001 |
| Lab test and medical imaging | **135.2 (881.0)** | **102.4 (568.0)** | 1,470.8 (2,028.9) | 178.9 (1906.8) | 0.2348 |
| Hospital bed fee | **19.4 (103.5)** | **13.4 (75.7)** | 1,082.0 (225.2) | 25.8 (119.2) | 0.0001 |

Cost data are presented as mean in Chinese Yuan (CNY); the US dollar equivalent can be calculated using the mean of 2015 exchange rates: US$1=CNY 6.2284. *****Reference group. SD: standard deviation. Costs were compared in Kruskal-Wallis; *P* values indicated overall significance of each comparison. Two separate significance testes were also used to compare mild group vs. severe group, and moderate group vs. severe group, respectively.

**Supplementary Table 4. Medical resource utilization by children under 14 years**

|  | **Mild**  **(n=364)** | **Moderate**  **(n=154)** | **Severe***  **(n=22)** | **Total**  **(n=540)** | **p-Value** |
| --- | --- | --- | --- | --- | --- |
| Severe exacerbation (%, yes) | 70(19.2%) | 31(20.1%) | 9(40.9%) | 110 (20.4%) | 0.049 |
| Control level (%, poor) | 130(35.7%) | 52(33.8%) | 9(40.9%) | 191(35.4%) | 0.784 |
| Outpatient rate (%) | 315 (86.5%) | 135(87.7%) | 14(63.6%) | 464(85.9%) | 0.009 |
| Inpatient rate (%) | 34(9.3%) | 18 (11.7%) | 7 (31.8%) | 59 (10.9%) | 0.004 |
| Utilization rate of (%) |  |  |  |  |  |
| OCS (%) | 5(1.4%) | 5(3.3%) | 7(31.8%) | 17 (3.2%) | 0.000 |
| ICS (%) | 211(58%) | 107(69.5%) | 17(77.3%) | 335(62%) | 0.015 |
| Systematic CS (%) | 44(12.1%) | 21 (13.6%) | 9(40.9%) | 74(13.7%) | 0.001 |

*****Reference group. OCS: oral corticosteroid use; ICS: inhaled corticosteroid; CS: corticosteroid use Chi-square tests/Fisher-exact tests were used to compared the differences in medical resource utilization. *P* values indicated overall significance of each comparison. Two separate significance testes were also used to compare mild group vs. severe group, and moderate group vs. severe group, respectively. Bold indicates values that are significantly different from the reference level.
